# Extracellular vesicles carry distinct proteo-transcriptomic signatures that are different from their cancer cell of origin

**DOI:** 10.1016/j.isci.2022.104414

**Published:** 2022-05-18

**Authors:** Tzu-Yi Chen, Edgar Gonzalez-Kozlova, Taliah Soleymani, Sabrina La Salvia, Natasha Kyprianou, Susmita Sahoo, Ashutosh K. Tewari, Carlos Cordon-Cardo, Gustavo Stolovitzky, Navneet Dogra

**Affiliations:** 1 Department of Pathology, Icahn School of Medicine at Mount Sinai, New York 10029, USA; 2 Department of Genetics and Genomics Sciences, Icahn School of Medicine at Mount Sinai, New York 10029, USA; 3 Department of Oncological Sciences, Icahn School of Medicine at Mount Sinai, New York, NY 10029, USA; 4 Department of Cardiology, Icahn School of Medicine at Mount Sinai, New York 10029, USA; 5 Department of Urology, Icahn School of Medicine at Mount Sinai, New York 10029, USA; 6 IBM T. J. Watson Research Center, Yorktown Heights, New York 10598, USA; 7 Sema4, Stamford, CT 06902, USA

**Keywords:** Cancer systems biology, Microenvironment, Transcriptomics

## Abstract

Circulating extracellular vesicles (EVs) contain molecular footprints—lipids, proteins, RNA, and DNA—from their cell of origin. Consequently, EV-associated RNA and proteins have gained widespread interest as liquid-biopsy biomarkers. Yet, an integrative proteo-transcriptomic landscape of EVs and comparison with their cell of origin remains obscure. Here, we report that EVs enrich distinct proteo-transcriptome that does not linearly correlate with their cell of origin. We show that EVs enrich endosomal and extracellular proteins, small RNA (∼13–200 nucleotides) associated with cell differentiation, development, and Wnt signaling. EVs cargo specific RNAs (RNY3, vtRNA, and MIRLET-7) and their complementary proteins (YBX1, IGF2BP2, and SRSF1/2). To ensure an unbiased and independent analyses, we studied 12 cancer cell lines, matching EVs (inhouse and exRNA database), and serum EVs of patients with prostate cancer. Together, we show that EV-RNA-protein complexes may constitute a functional interaction network to protect and regulate molecular access until a function is achieved.

## Highlights

• Extracellular vesicles (EVs) enrich unique RNA & proteins compared to cell of origin
• EVs enrich small coding/noncoding RNAs (13–200 nt) associated with cell development
• Blood EVs of patients with prostate cancer reveal immune regulation function
• EVs RNA-proteins are inter-related and show functional interaction network

## Graphical abstract

**Figure:**
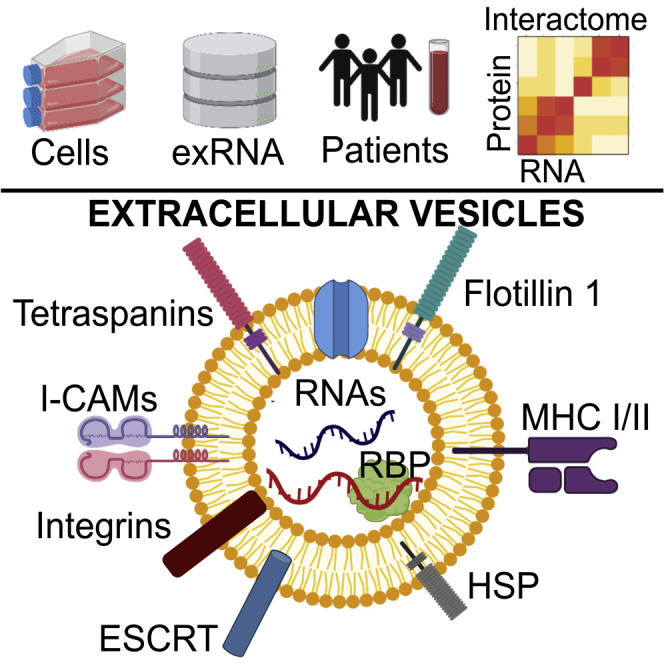

## Introduction

Emerging evidence suggests that circulating EVs contain molecular footprints—lipids, proteins, metabolites, RNA, and DNA—from their cell of origin for intercellular communication (Kowal et al., 2014; Simpson et al., 2009; Valadi et al., 2007). Consequently, EVs are increasingly recognized as key players in the signal transduction, priming of tumor microenvironment, and metastasis (Gaglani et al., 2021; N. Dogra et al., 2020), mainly through cellular crosstalk and vesicle trafficking (Hoshino et al., 2015; Kamerkar et al., 2017; Krishn et al., 2020). First described in the 1980s (Johnstone et al., 1987; Trams et al., 1981), exosomes are extracellular nanovesicles of endocytic origin, which are formed by the inward budding of a late endosome, also known as multivesicular body (MVB) (Kowal et al., 2014; Raposo and Stoorvogel, 2013; Simpson et al., 2009). Subsequently, MVBs fuse with the plasma membrane resulting in the release of exosomes into the extracellular environment (Johnstone et al., 1987; Trams et al., 1981). Nevertheless, exosomes are merely a subset of secretory EVs, other particles include—but are not limited to—apoptotic, micro-, onco-vesicles (Kalluri and LeBleu, 2020; Kowal et al., 2014; Raposo and Stoorvogel, 2013), and exosome-like enveloped viruses (Dogra et al., 2021). Accumulating evidence show that while all EVs carry molecular footprints from their cell of origin, exosomes selectively package proteins, nucleic acids, and do not appear enriched in cellular debris (Kowal et al., 2014; Lotvall et al., 2014). In understanding the complete cargo of EVs, recent studies have successfully utilized proteomic and transcriptomic technologies for their molecular analyses and have compiled comprehensive databases accordingly (Bellingham et al., 2012; Murillo et al., 2019; Simpson et al., 2009). “Extracellular RNA (exRNA) Atlas” and “Vesiclepedia” are two such comprehensive databases (Kalra et al., 2012; Murillo et al., 2019; Rozowsky et al., 2019).

Currently, the majority of published EV literature is analyses of either the proteomic or the transcriptomic signatures alone (Keerthikumar et al., 2015; Mathivanan et al., 2010; Simpson et al., 2009). While numerous studies have investigated donor cells and their EVs’ content, inter-related functionalities between EV proteins and RNAs remain unexplored (Eirin et al., 2017). This is mainly due to a lack of proteomic and transcriptomic analyses of matching donor cells and their EVs. An integrative proteo-transcriptomic analysis of EVs will not only reveal the mutual regulation between RNA and proteins but will also provide key insights into their applications as therapeutic and non-invasive next-gen liquid-biopsy procedures (N. Dogra et al., 2020; Smith et al., 2018).

**Figure 1:**
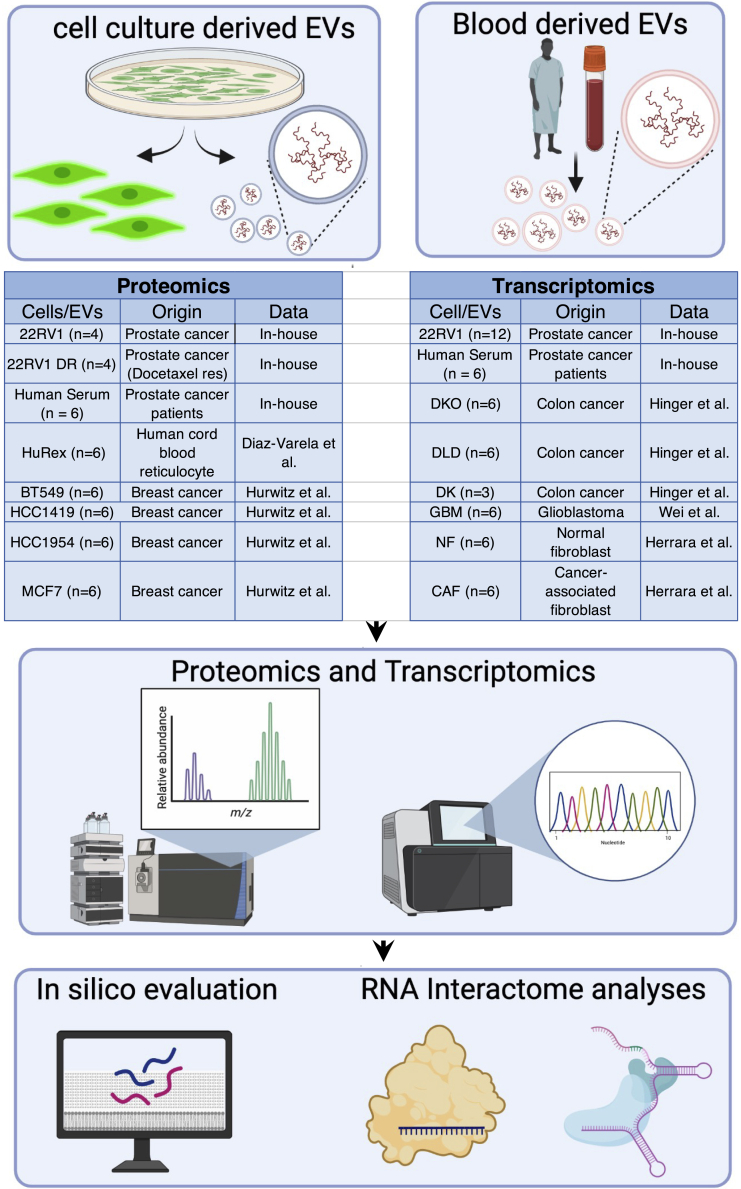
Overview of the experimental design and data analysis Extracellular vesicles (EVs) are isolated from cell culture media and serum of patient with prostate cancer (PCa). Subsequently, the proteo-transcriptome of cells and their EVs are characterized. Detailed information regarding the proteomic and transcriptomic datasets used in this study is provided in the respective tables. In addition to in-house data generation, external data are curated from following studies (Hurwitz et al., 2016, Diaz-Varela et al., 2018, Hinger et al., 2018, Wei et al., 2017 and Herrera et al., 2018).

In this study, we have generated and curated comprehensive datasets of proteomics and transcriptomics from a total of 12 cancer cell lines, their EVs, and serum EVs of patients with prostate cancer (A detailed description of datasets is provided in Figure 1). To identify EV-specific molecular signatures, we compared the RNA and protein profiles of EVs with their donor cells and assigned them respectively to individual subcellular locations according to Gene Ontology (GO) annotation. Next, we asked whether EV’s protein and RNA cargo are inter-related and may converge to achieve the same biological function. To address this, we utilized RNA-interactome of the protein and RNA cargo of EVs and examined for functionality, mutual regulations, and distinct cellular pathways. Finally, we put forth a comprehensive model that integrates the proteomic and transcriptomic signature of EVs, providing a conceptual advance in the development of next-generation clinical assays via a multi-omic (proteomic and transcriptomic) approach for liquid biopsy in numerous diseases.

## Results

Our study has generated and curated comprehensive datasets of proteomics and transcriptomics from a total of 12 cancer cell lines and their EVs, and serum EVs of patients with prostate cancer (A detailed description of datasets is provided in Figure 1). The three datasets have been labeled as 1) A “*transcriptomics*” dataset composed of small RNAseq of six cancer cell lines and their EVs. 2) A “*proteomics*” dataset composed of mass spectrometric analyses of six cancer cell lines and their EVs. 3) A distinct “*human serum EV*” cohort composed of proteomics and transcriptomics from six serum EVs of patients with prostate cancer.

### Characterization of extracellular vesicles

**Figure 2:**
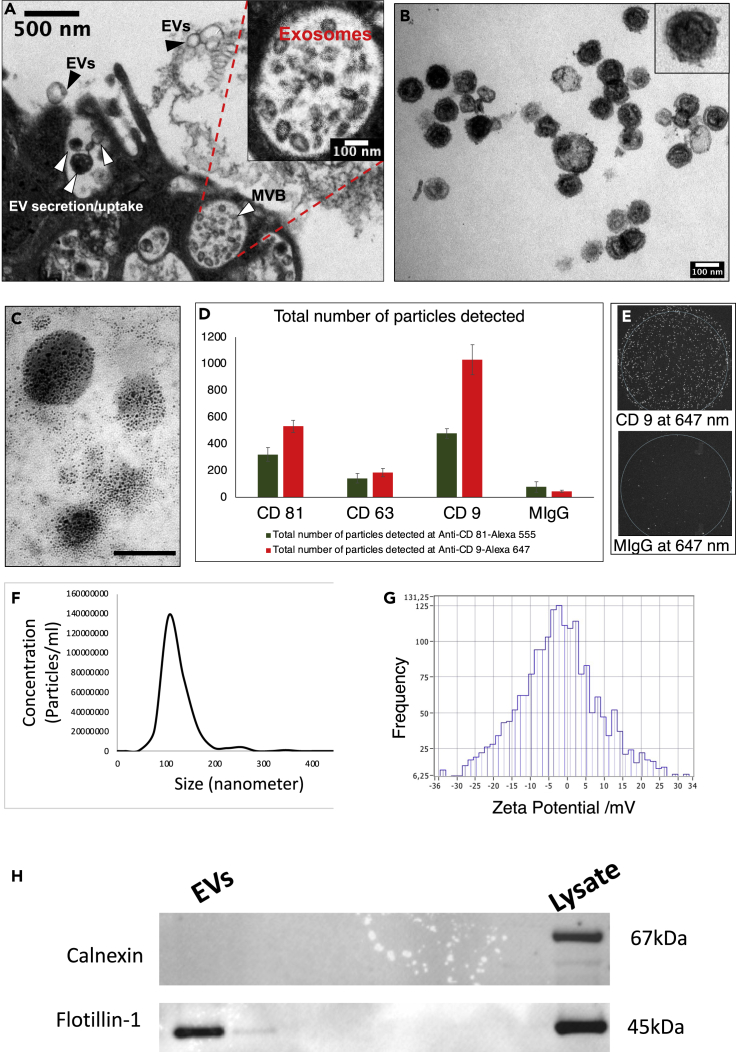
Characterization of cancer-cell-culture-derived EVs (A) Transmission electron microscopy (TEM) image of EVs released from prostate cancer cells. (B) TEM of isolated EVs. (C) Canonical markers immunogold (6nm)-labeled CD81 shows specificity to the vesicle surface. The scale bar is 100nm. (D) Immunofluorescence co-localization analyses of EVs confirmed the presence of canonical tetraspanins on the vesicle surface. Mouse IgG antibody yielded a minimum signal. (E) Optical image of immunofluorescence co-localization analyses shows EVs specificity to CD9 but not to mouse IgG. (F) Nanoparticle tracking analysis (NTA) shows size and concentration of isolated EVs. (G) Zeta potential of EVs shows moderate colloidal stability between the EVs and their surrounding fluid environment. (H) Western blot analyses of EVs and cell lysate.

First, we conducted a comprehensive quality control analysis of EVs to characterize their size, shape, morphology, and canonical markers. These analyses comprise transmission electron microscopy (TEM), immunogold TEM, nanoparticle tracking analyses (NTA), zeta potential analyses, and multicolor immunofluorescence co-localization analyses (nanoview). To characterize the morphology, donor cells and their EVs were examined under a high-resolution TEM revealing the release of typical cup-shaped EVs from 22RV1 prostate cancer cells (Figure 2A). We observed two mechanisms of EV secretion: 1) vesicles of endocytic origin (exosomes) secreted via fusion of MVBs with the plasma membrane and 2) secretion via budding (exocytosis) from the plasma membrane. Notably, the first mechanism displayed consistent round and cup-shaped ∼80 nm vesicles, while the second mechanism displayed relatively larger and heterogeneous, ∼50–250 nm vesicles. The isolated EVs were observed under a TEM, revealing vesicles of ∼80–100 nm sizes (Figure 2B). The immunogold TEM captured CD81 (a canonical exosome marker) antibody attached to 6 nm colloidal gold particles (Figure 2C). Furthermore, the immunofluorescence co-localization analyses (see STAR Methods) of EVs revealed three vastly studied canonical tetraspanin exosome markers CD81, CD9, and CD63 on vesicles surface (Figure 2D). As expected, all three exosomal tetraspanins were enriched on the vesicle’s surface, confirming the presence of canonical markers for exosomes. In contrast, the mouse IgG was non-specific to the EVs and displayed no signal for tetraspanin proteins (Figures 2D and 2E lower panel). The EVs’ zeta potential ranged from −30 to +30 mV (highest at −3 mV) demonstrating moderate colloidal stability between the EVs and their surrounding fluid environment (Figure 2G). Taken together, our EVs are typically within the range of ∼50–200 nm in diameter while the majority are enriched for a median of ∼80 nm sized particles (Figures 2B and 2C). EVs enrich canonical tetraspanins CD81/CD9/CD63 (Figures 2C–2E and 2H) for exosomes and demonstrates moderate colloidal stability (Figure 2G).

### EVs enrich for protein signatures that are different from their cancer cell of origin

To characterize the proteomic landscape, we conducted liquid chromatography with tandem mass spectrometry (LC-MS/MS) of donor cells and their EVs. To reliably identify a protein, positive identification was set at 5% protein false discovery rate (FDR) and 1% peptide FDR. Also, at least two unique spectra have to be identified per protein. Scaffold Proteome Software was used for post-database search processing. All samples were analyzed in duplicates. As a result, 422 proteins for EVs and 5630 proteins for cells passed our filtering criteria. Notably, the top 5% of EV proteins were molecules of endosomal sorting complex required for transport (ESCRT), endosomal origin (clathrin), multivesicular body (MVB12A), membrane trafficking (RAB proteins and annexins), cytoskeletal (actin, tubulin, and myosin), heat shock proteins (HSC70, HSP71, and HSP90), and exosomal markers of endocytic origin (ALIX) and TSG101. These findings are in accordance with literature, as ESCRT and other endocytic machinery proteins are enriched in exosomes (Kalra et al., 2012; Simpson et al., 2009).

**Figure 3:**
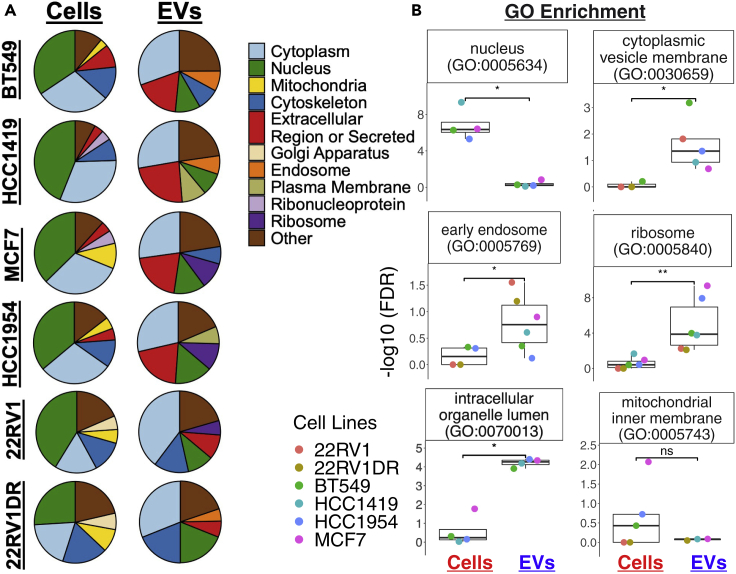
EVs enrich for unique protein signatures that are different from their cancer cell of origin (A) Comparative analyses of proteomic data of EVs and their cancer cell or origin. Subcellular location of donor cancer cell proteome and EVs show that EVs enrich cytoplasmic, extracellular, and endosomal proteins. (B) Gene Ontology enrichment of proteins in cells and EVs. EVs enrich endosomal, cytoplasmic, and ribosomal proteins. Significance is demonstrated with following annotation: ns – not significant, ∗ - P<= 0.05, ∗∗ - P<=0.01. FDR are the adjusted p values using false discovery rate correction for multiple testing.

Next, we utilized the large proteomic dataset from the Vesiclepedia database, which is a compilation of data from over ∼1300 studies from EVs (Kalra et al., 2012). A comparison of our EV proteomic data with the Vesiclepedia database displayed an overlap of ∼94% (396 proteins) of the EVs proteins in our study relative to those in the Vesiclepedia, leaving only ∼6% (26) of the EV proteins unique to our study (Figure S1A). These results confirmed that our EV isolation methods and proteomic analyses were consistent, reproducible, and reliable with respect to the other ∼1300 studies present in the Vesiclepedia database. Finally, we compared the proteomics of donor cells with their EVs (Figure S1B). Of note, ∼22% (78) of the proteins were unique to the EVs and were not detected in their donor cells, while ∼78% (344) of the EVs proteins displayed an overlap with their donor cell’s proteins. To compare the subcellular location, both donor cells and EV-associated proteins were assigned to their respective locations according to Gene Ontology (GO) annotations (Figures 3A and 3B) using the Enrichr toolkit adjusted for multiple testing p < 0.01 (FDR<0.05)(Kuleshov et al., 2016). These analyses revealed that donor cells primarily contain nuclear proteins, while their EVs were predominantly enriched cytoplasmic proteins (Figures 3A, 3B, S1A, and S1B). Also, EVs are enriched with 2-4 times more cytoskeletal and extracellular proteins than their donor cells (FDR<0.05). In contrast, the donor cells carry 3–10 times more mitochondrial and nuclear proteins, respectively (FDR<0.05). To ensure that our analyses provide a global proteome of EVs in an unbiased manner, we curated external proteomic datasets of four additional breast cancer cell lines (details provided in Figure 1) and human cord blood reticulocyte-derived (HuRex) EVs and human serum EVs (Diaz-Varela et al., 2018). Similar to our inhouse proteomic analyses for EVs, these analyses displayed predominant enrichment of cytoplasmic, endosomal, and extracellular proteins in EVs (FDR<0.05) (Figures 3A, 3B, S1E and S1F).

Overall, we demonstrate that the proteomic profile of EVs is comprised of numerous proteins exclusively enriched in EVs but not in their donor cells. Furthermore, the integration of external datasets with our in-house data solidified our findings, hence making our statement independent of the cell types, EV isolation methods, site of experimentation, site of data generation, and methodologies applied for mass spectrometry.

### Transcriptomic analyses (small RNA sequencing) of EVs and donor cells

**Figure 4:**
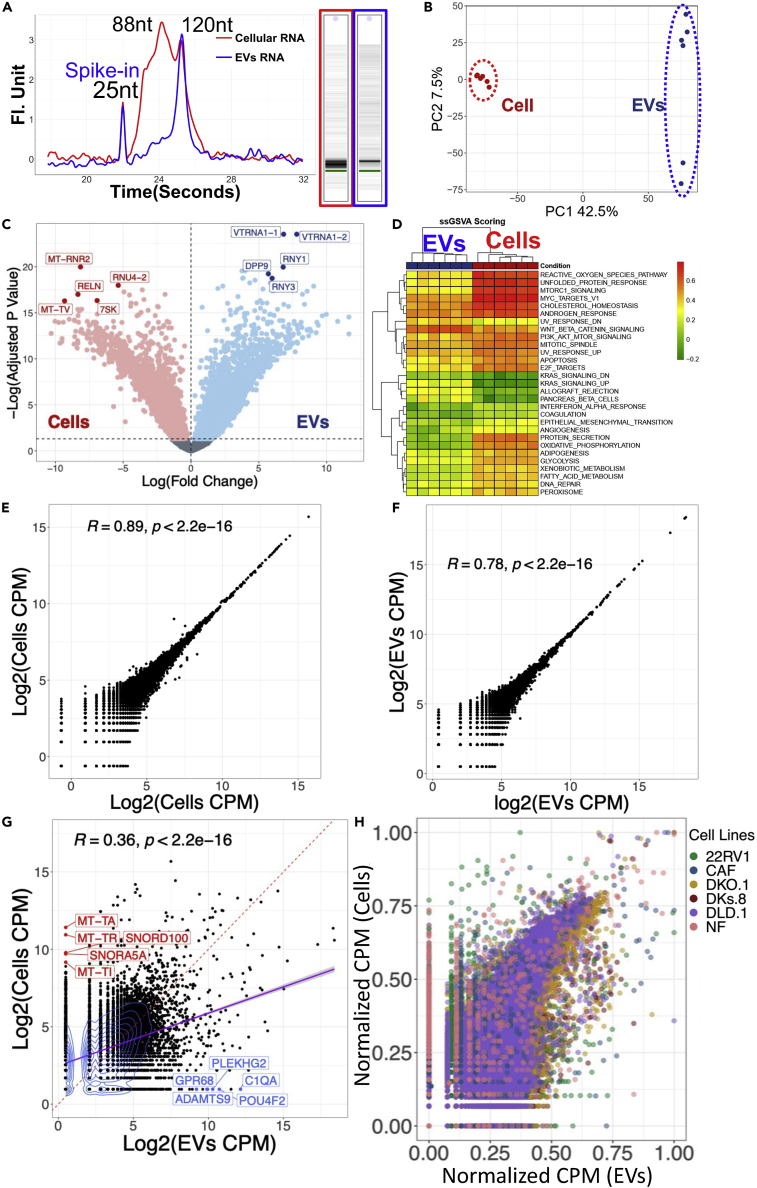
EVs enrich for unique RNA signatures that are different from their cancer cell of origin (A) Capillary electrophoresis analyses of small RNA from donor cells (22RV1) and their EVs. (B) Principal component analysis (PCA) of the RNA between cells and EVs shows distinct RNA in EVs. (C) Volcano plot showing differential enrichment of RNA between Cells and EVs. (D) Hallmark cancer pathways identified in EV and cellular RNA. (E) Two biological runs yielded a high correlation of cell versus cell RNAseq. (F) Two biological runs yielded a high correlation between EVs vs EVs RNAseq. (G) Comparison between donor cells versus their EVs RNA does not yield a high correlation. (H) A normalized gene expression of six different cell lines for donor cells versus their EVs RNAseq. In all studies, EVs enriched for distinct RNA, which were predominantly different in their cell of origin.

Recent studies have shown that EVs contain diverse small RNA subtypes, ranging between ∼20 and 200 nucleotides (nt) (Srinivasan et al., 2019; Wei et al., 2017). However, the types of RNA packaged inside EVs remain a matter of intense debate (Srinivasan et al., 2019; Valadi et al., 2007; Wei et al., 2017). To assess and compare the precise length of RNA of donor cells and their EVs, we extracted total RNA and characterized it through capillary electrophoresis using two separate analyses kits: Pico and small RNA kits for bioanalyzer (Figures 4A and S2). These analyses revealed that although both donor cells and their EVs displayed small RNAs, their lengths are distinct. For instance, a major peak around ∼100 nt is predominantly present in EVs, while cells contain additional small RNA peaks at ∼80 nt and ∼50 nt (Figure 4A). EVs contain major peaks at ∼60 and 100 nt, which may correspond to tRNA and small nuclear RNA (snRNA) (Figure S2). These observations compelled us to survey the current small RNA landscape under 200 nt (Murillo et al., 2019; Wei et al., 2017). Among the small RNAs, miRNAs (∼21 nt), siRNAs (∼20–25bp), tRNAs (∼60–95) nt, 5S rRNAs (∼120 nt), Y RNA (80–120 nt), and snRNAs (∼150 nt) are potential candidates that may be present in EVs (Boivin et al., 2019). To address the different subtypes of RNAs in EVs and cells, we proceeded with cDNA library preparation followed by total small RNA sequencing. A detailed analysis of the transcriptomic profiles is discussed in the section below.

### EVs enrich for unique RNA signatures that are different from their cancer cell of origin

To identify the entire payload of the donor cells and their EVs’ RNA, we conducted total small RNA sequencing and investigated their gene expression profiles. We identified over ∼20, 000 distinct RNA molecules in EVs and donor cells. The principal component analysis (PCA) showed that the molecular profiles were uniquely distinct between cells and EVs with a total of 50% variance explained by PC1 and PC2 (Figure 4B). The variance between EVs and their cell of origin indicates that the most abundant transcripts in cells were different from those in EVs. To understand the landscape of RNA within cells and their EVs, we performed a differential expression analysis using mixed linear models revealing over 8000 differentially expressed RNAs with a log fold change up to 10 times (FDR<0.05) (Figure 4C). The observations of gene biotype revealed that distinct cargo types are packaged with effector and regulatory RNA molecules, while cells predominantly contain mtRNAs and rRNAs (Figure 4C).

Of note, numerous RNA molecules were only detected in the EVs, not in their donor cells (Figure 4C). EVs exclusively contained PLEKHG2 (nucleotide-binding protein), GPR68 (a proton sensing G protein-coupled receptor 68), POU4F2, ADAMTS9, and Let-7 microRNA precursor, which are involved in cell development, signal transduction, and cancer metastasis (Schaefer et al., 2009; Shurtleff et al., 2017; Valadi et al., 2007). Consistent with these observations, the results of gene set enrichment analyses of hallmark cancer pathways showed that cells were enriched for cellular damage response and that these signatures were retained in EVs for pathways such as MYC targets or androgen response (Figure 4D)(Gross et al., 2012; Miyamoto et al., 2015; Zhang and Wrana, 2014). Wnt beta-catenin and KRAS signaling was enhanced in EVs, suggesting EVs’ involvement in promoting cancer progression and metastasis (Figure 4D). This observation, in accordance with multiple studies, demonstrates the significance of Wnt pathways in cell regulatory processes including cell proliferation, stem cell differentiation, and migration (Murillo et al., 2019; Smith et al., 2018). It is important to discuss that our RNAseq studies were reproducible as replicates of cell versus cell (two biological replicates) and EVs versus EVs yielded high correlation, Rho = ∼0.9 and ∼0.8 (p < 0.05), respectively (Figures 4E and 4F). In contrast, the correlation between donor cells and their EVs yielded Rho = 0.36, p < 0.05 (Figure 4G), indicating there was little correlation between EVs and their donor cells.

Finally, to ensure that our analyses are independent of the cell types, EV isolation methods, site of experimentation, site of data generation, and methodologies applied for RNAseq (cDNA library prep and downstream analyses), we curated external transcriptomic datasets of five additional cell lines (details provided in Figure 1). These combined analyses of cell lines and their EVs demonstrated that irrespective of the cell type and other aforementioned conditions, EVs enrich for distinct RNA signatures that do not correlate with their cell of origin (Figure 4H). A detailed list of the top 50 RNA enriched in EVs and their cell of origin from six cell lines is provided (FDR<0.05 & abs(LogFC) > 1) (Table S4).

### Proteo-transcriptomic analyses of EVs from serum of patient with prostate cancer reveal their mRNA binding and immune regulation function

**Figure 5:**
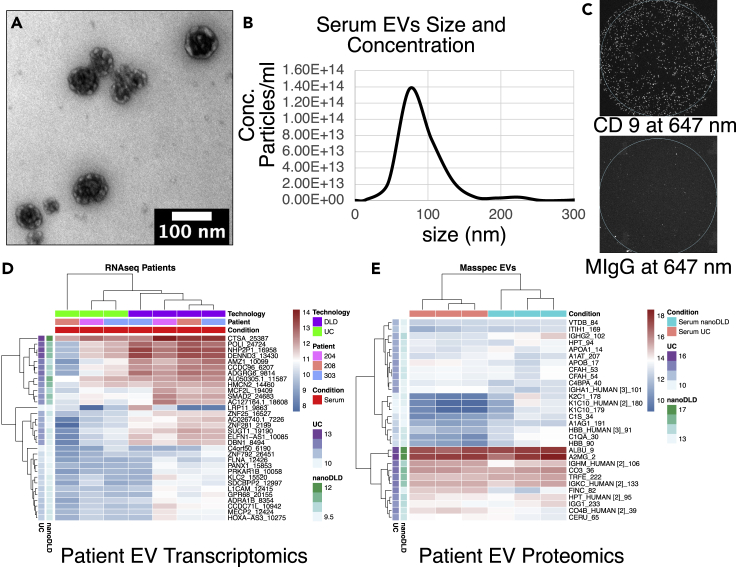
Proteo-transcriptomic analyses of EVs from serum of patient with prostate cancer reveal distinct RNA and protein signatures (A) TEM image of UC isolated EVs. (B) A size and concentration analyses of serum EVs show distribution of vesicles (50–150 nm). (C) Immunofluorescence co-localization analyses of serum EVs confirmed the presence of canonical tetraspanin CD9 on vesicle surface. Optical image of immunofluorescence co-localization analyses shows EVs specificity to CD9 but not to mouse IgG. (D) Top 30 proteins detected in serum EVs. (E) Top 30 RNA detected in serum EVs.

**Figure 6:**
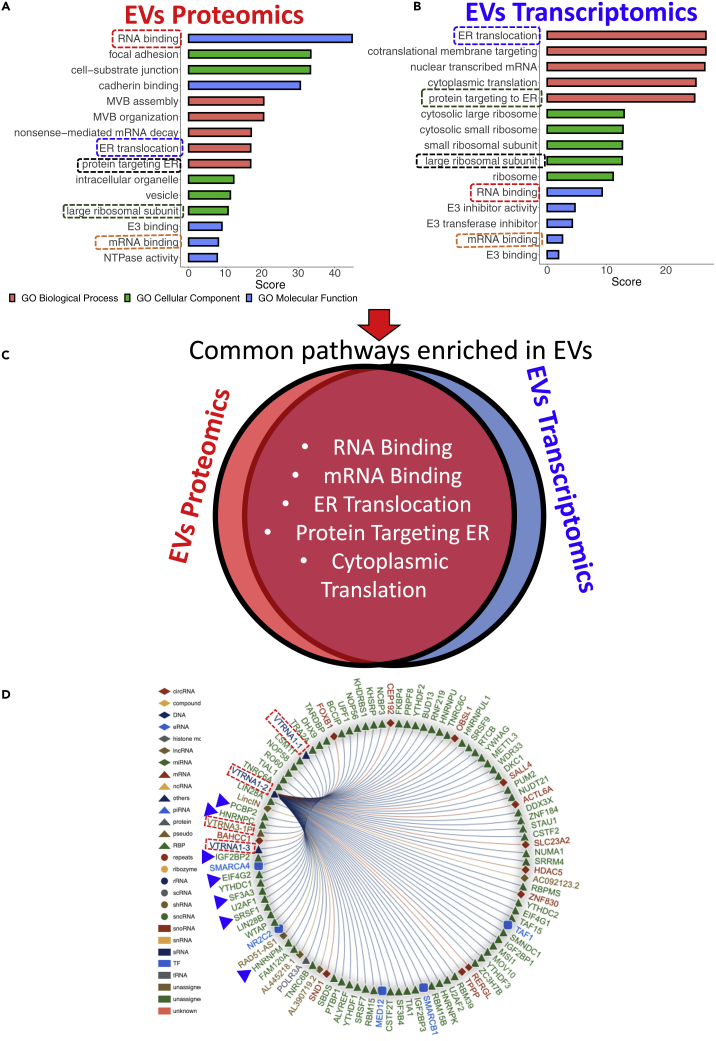
Integrative proteo-transcriptomic analyses of EVs reveal enrichment of RNA-proteins and RNA-RNA complexes (A) Top enriched pathways identified in proteomic analyses of EVs. (B) Top enriched pathways in transcriptomic analyses of EV. (C) Overlapping proteo-transcriptomic pathways enriched in EVs. (D) A representative RNA interactome analysis shows RNA (red box) and their target proteins identified in EVs.

Encouraged by our studies from cells and EVs, we isolated EVs from the serum of six patients with prostate cancer and investigated their combined proteo-transcriptome. To understand EVs’ content irrespective of the isolation method used (Murillo et al., 2019), we used two separate technologies (buoyant density-based UC, and size-based nanoDLD (N. Dogra et al., 2020; Smith et al., 2018)) to isolate EVs followed by their proteo-transcriptomic analyses. First, we characterized serum EVs for their size, shape, morphology, and canonical marker of EVs. Similar to cell culture derived EVs, TEM showed round, cup-shaped morphology for serum EVs (Figure 5A). The EVs ranged from ∼50 to 150 nm in size and carried CD9 on their surface (Figures 5A–5C). Rigorous characterization of UC and nanoDLD-isolated EVs from various biofluids has been presented in our previous publications (Murillo et al., 2019; Smith et al., 2018). Our proteo-transcriptomic analyses from serum EVs displayed specific signals, highlighting the presence of an EV-specific signature (FDR<0.05 & abs(logFC) > 1) (Figures 5D and 5E). The serum EV signatures from RNAseq analysis (FDR<0.05 & abs(logFC) > 1) are associated with protein binding, epithelial cell proliferation, post-translational protein regulation, and regulation of synaptic plasticity (Figure 6A). The pathway enrichment results (FDR<0.05) of the mass spectrometry are highly correlated with the regulation of complement cascade or immune response, and processes (Figure 6B). Overall, similar to the *in vitro* cell culture, serum EVs carry molecular signatures that implicate protein and RNA regulation.

### Integrative proteo-transcriptomic analyses of EVs reveal enrichment of RNA-proteins and RNA-RNA complexes

Intracellular amino acids and their complementary nucleotides form protein-RNA complexes that protect and regulate transcripts through their life cycle until a function is achieved (Corley et al., 2020). However, the current landscape of EV encapsulated RNA-protein complexes and their inter-relationship remains uncharted territory. Here, we used RNA interactome database to identify EV-enriched RNA/proteins and their target RNA-protein and RNA-RNA interactions (Lin et al., 2020). We discovered that transcription factor YBX1 and its target RNA (RNY3, a Y RNA class) were significantly enriched (FDR<0.05) in our proteo-transcriptomic analyses of EVs (Table S1). Vault RNAs (vtRNA 1-1/2/3) were enriched in EVs, and their complementary binding proteins (IGF2BP2 and SRSF1) were enriched in our proteomic analyses (Figure 6D). We also identified various RNA-RNA complexes in EVs. We found that Let-7 (a predominant RNA in EVs) and its associated RNA (SPEG) and protein (SRSF2) were enriched in EVs (FDR<0.05). Finally, functional annotation analyses (FDR<0.05) revealed that RNA binding, protein translation, and gene expression are the major overlapping molecular pathways between the EV’s RNA and proteins, implicating a coordinated mechanism between EVs and their donor cells (Figure 6C).

## Discussion

**Figure 7:**
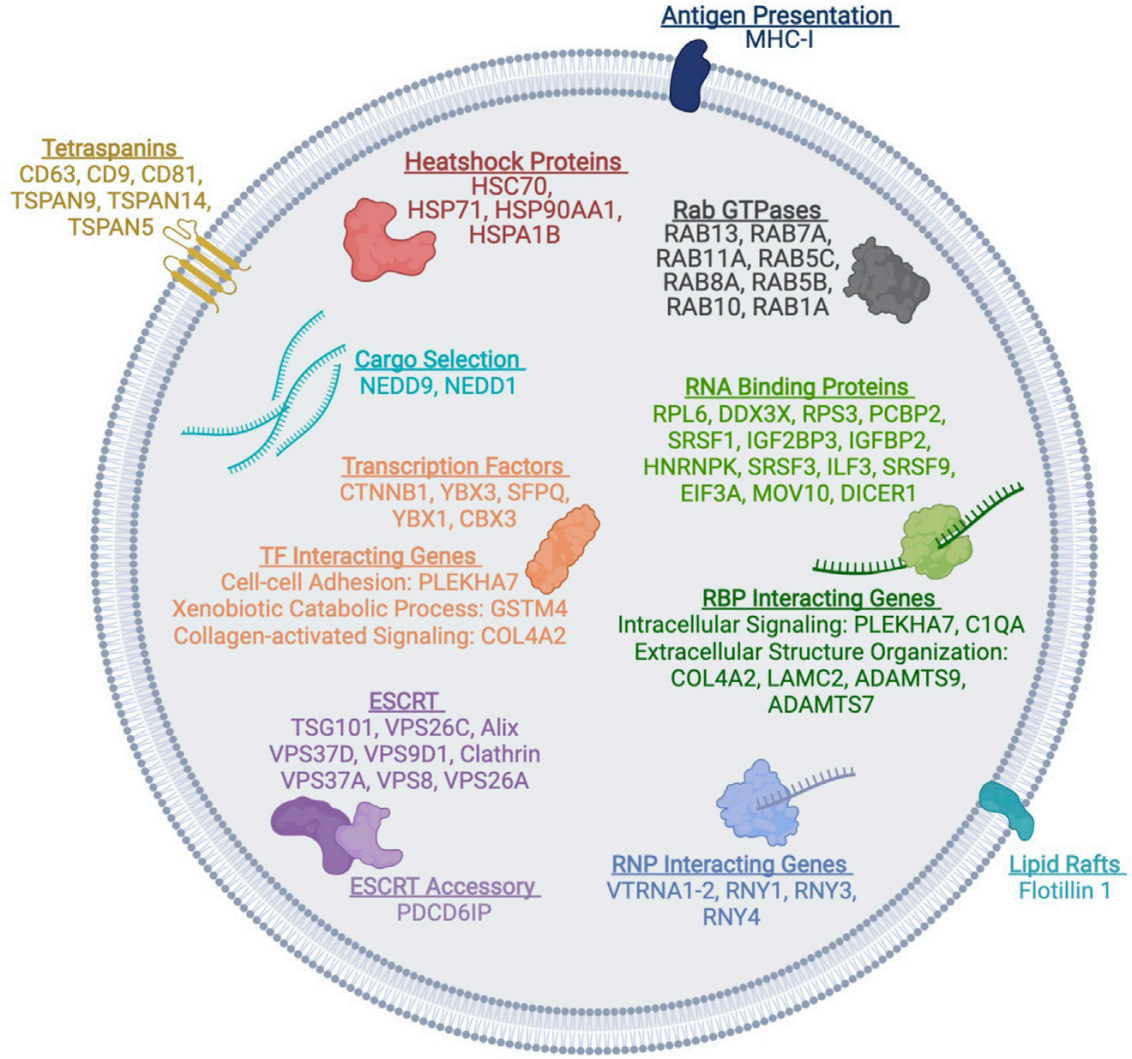
A graphical representation of integrative proteo-transcriptomic composition of EVs Top enriched proteins and their target RNA enriched in EVs.

This study provides a comprehensive analysis of the proteo-transcriptome of EVs from cancer cells and human serum (Figure 7). We show that EVs encapsulate distinct RNA and protein cargo that is predominantly different from their cancer cell of origin. Of note, many RNA and proteins were exclusively enriched in the EVs but not their cell of origin. EVs are enriched for endosomal, multivesicular body proteins, membrane trafficking, ESCRT, and exosomal marker proteins. Our subcellular compartment enrichment analyses reveal that EV cargo 4–6 times more cytoskeletal, endosomal, extracellular, and cytoplasmic proteins. The RNA characterization revealed that EVs carry a range of small RNA between ∼15 and 200 nt, which display miRNAs, siRNAs, tRNA, Y RNA, 5S rRNAs, and snRNAs. Although there are no established canonical RNA markers for EVs, a recent extracellular RNA (exRNA) study investigated and compared exRNA cargo types in over 5000 human samples using different isolation methods and biofluids (Murillo et al., 2019). This independent analysis found that our isolation methods (UC and nanoDLD chip technology(Smith et al., 2018) specifically isolate low- and high-density vesicles (cargo type 1 & 4, respectively) with minimum contamination from the lipoproteins and argonaute proteins (Murillo et al., 2019).

To ensure that our findings provide an unbiased outcome that is not dependent upon the cell of origin, EV isolation methods, site of experimentation, site of data generation, and protocols applied for RNAseq and mass spectrometry, we curated external datasets of 10 additional cell lines (details provided in Figure 1). These analyses revealed that irrespective of the cell type and other aforementioned conditions, EVs enrich for distinct proteo-transcriptomic signatures that do not linearly correlate with their cell of origin.

Our results are supported by several independent studies that have indirectly shown that EVs proteins and/or RNA were differentially expressed compared to their cell of origin (Kalra et al., 2012; Valadi et al., 2007; Wei et al., 2017). Considering that the diameter of EVs is ∼50–200 nm, size may play a major role in the packaging of RNA and larger RNAs are likely beyond the EVs’ packaging capacity (Valadi et al., 2007; Wei et al., 2017). This observation is supported by a recent study on glioblastoma derived EVs, which showed that ∼3000 nt RNAs were not present in the small EVs but were abundant in the donor cells and their microvesicles (Wei et al., 2017). We hypothesize that these observations can be reasoned via the following mechanisms: 1) The structural integrity of EV-encapsulated RNA and proteins may be enhanced as a consequence of RNA-RNA or RNA-protein complex formation, while separating such molecules from cytoplasmic nucleases/proteases, hence enhancing their half-life and overall stability. 2) The delivery and disposal aspect of EVs may implicate a coordinated effort of enrichment of molecules in the EVs while lowering their abundance in the donor cells. Given the immense excitement that EVs research has generated in recent years, our findings are significant for the following major reasons:

### Significance of EVs enriched proteins and RNA

The observation that EVs are enriched for exclusive molecules other than the most abundant cargo in their cell of origin has immense significance with respect to mass EV production for gene delivery and other therapeutic applications. Currently, several industrial and academic institutions are attempting to overexpress RNAs and proteins of interest in donor cells given the assumption that secreted EVs will enrich the most abundant molecule. In fact, the inter-relationship of EV-associated RNA and proteins has been presumed to be linearly correlated with their cell of origin. However, based on our studies, we find that the most abundant molecules in the cells may not be enriched in EVs. These findings suggest that alternate strategies, similar to liposomes encapsulation (Dogra et al., 2015, 2016, 2019), must be followed for enriching cargo inside the EVs (Dogra et al., 2019; Haraszti et al., 2018).

### Significance of EVs enriched RNA-proteins complexes

We show that EVs enrich proteins and RNA that are functionally inter-related using robust statistical tests and data modeling strategies. These findings are in accordance with previously published studies that have shown intracellular associations of several transcription factors, other proteins, and RNA. For instance, RNY3 interacts with transcription factor YBX1, which helps block access to the RNA (Corley et al., 2020; Lin et al., 2020; Shurtleff et al., 2017). A recent study showed YBX1 and its association with mir-223 in exosomes (Shurtleff et al., 2017). We show that enrichment of various RNAs (RNY3, vtRNA, and MIRLET-7) and their complementary proteins (YBX1, IGF2BP2, and SRSF1/2) in EVs may target distinct cellular pathways that converge to achieve the same biological goals (Figure 7). Furthermore, these analyses reveal that RNA-protein complex could provide a potential functional interaction network inside EVs to protect and regulate access to the RNA. Thus, EV’s mRNA, miRNA, and proteins may be connected to achieve the same regulatory function.

In summary, we report that EVs enrich for distinct RNA and protein signatures that do not linearly correlate with their cell of origin. Our integrative proteo-transcriptomic analyses suggest that RNA-protein complexes may constitute a functional interaction network inside EVs to protect and regulate access to EV-RNA, until a function is achieved. This is of major clinical significance in the potential use of the integrative multi-omic proteo-transcriptomic platform that would further enhance the conceptual advance and diagnostic performance of diseases via liquid biopsy.

### Limitations of the study

The literature of nanosized EVs is still developing and today many vesicles fall under the vast canopy of EVs. Several nanosized (<100nm) EVs are still waiting to be discovered. We hypothesize that there are different subsets of nano EVs and many will be discovered in the near future. This means that the EV landscape may change, and some extracellular particles or vesicles may directly correlate or anticorrelate with their cell of origin, which remains to be seen in the near future. Furthermore, we acknowledge the low number (n = 6) of patient samples. Despite these limitations, we demonstrate proof-of-concept study and our ability to conduct reproducible and integrative proteomic-transcriptomic analyses of 12 cancer cell lines and patient blood-derived EVs. A potential limitation of our and previous studies found in the literature is the sensitivity to detect low quantities of protein and RNA from EVs; however, we used our published ultra-low input approach for these studies and robust statistical tests to account for false positives (Murillo et al., 2019; N. Dogra et al., 2020).

## STAR★Methods

### Key resources table

**Table:** 

REAGENT or RESOURCE	SOURCE	IDENTIFIER
**Antibodies**		

CD81	Abcam	PRID: ab239687
Calnexin	Abcam	PRID: ab22595
Gold conjugated secondary antibody	Abcam	PRID: ab105285
Flotillin (anti-Flot-1)	Abcam	PRID: ab133497

**Biological samples**		

Serum samples from prostate cancer patients	Icahn School of Medicine at Mount Sinai; Department of Urology	IRB, GCO # 06-0996, 14-0318
Human prostate cancer cell line 22RV1	American Type Culture Collection	ATCC CRL-2505

**Chemicals, peptides, and recombinant proteins**		

RPMI 1640 cell culture medium	GIBCO	
Bovine serum albumin in phosphate buffer saline	Sigma Aldrich	
3% Glutaraldehyde	Sigma Aldrich	
Osmium tetraoxide	Sigma Aldrich	
Acid-phenol	Invitrogen	Cat# 4478545
Chloroform	Invitrogen	Cat# 4478545
Lysis Buffer (2% SDS/1X protease inhibitor/0.1M Ambic)	Invitrogen	Cat# 4478545

**Critical commercial assays**		

Total EV RNA and Protein Isolation Kit	Invitrogen	Cat# 4478545

**Deposited data**		

Raw and normalized 22RV1 gene and protein counts	This manuscript	GSE123736
Human reference genome Ensembl GRCh38.p13	Ensembl	https://useast.ensembl.org/Homo_sapiens/Info/Annotation
Human breast cancer cell lines EV and cell proteomic counts	Hurwitz et al., 2016	PMID: 27894104
Human colon cancer cell lines EV and cell gene counts	Hinger et al., 2018	PMID: 30332650
Human normal and cancer-associated fibroblasts EV and cell gene counts	Herrera et al., 2018	PMID: 30075793
Human reticulocyte derived EV protein counts	Diaz-Varela et al., 2018	PMID: 30232403
Human glioblastoma cell derived 7.6572mmEV gene counts	Wei et al., 2017	PMID: 29074968

**Software and algorithms**		

QIAGEN Ingenuity Pathway Analysis	QIAGEN	https://digitalinsights.qiagen.com/products-overview/discovery-insights-portfolio/analysis-and-visualization/qiagen-ipa/?cmpid=QDI_GA_IPA&gclid=CjwKCAjwgr6TBhAGEiwA3aVuIS7UzgcUf8gTC8ad6uXMnBwZEeEb_lhG88SStmpLMASvAUl0tbI9tBoCJeAQAvD_BwE
FeatureCounts read summarization function	Subread	http://subread.sourceforge.net/
bowtie aligner (version 2.5.4b)	bowtie	http://bowtie-bio.sourceforge.net/bowtie2/index.shtml
R version 4.1.1	R Core Team	https://www.R-project.org/.
Proteome Discoverer software (version 2.1)	Thermo fisher scientific	https://www.thermofisher.com/us/en/home/industrial/mass-spectrometry/liquid-chromatography-mass-spectrometry-lc-ms/lc-ms-software/multi-omics-data-analysis/proteome-discoverer-software.html
Qlucore Omics Explorer software	Qlucore	https://qlucore.com/
Code availability	Github	https://github.com/chentytina/22RV1_EV

### Resource availability

#### Lead contact

Further information and requests for resources and reagents should be directed to and will be fulfilled by the lead contact, Dr. Navneet Dogra (navneet.dogra@mssm.edu).

#### Materials availability

This study did not generate new unique reagents.

### Experimental model and subject details

#### Patient recruitment and sample collection

The details of the IRB/oversight body that provided approval or exemption for the research described are given below: Institute Review Board approved protocols (GCO # 06-0996, 14-0318, and surgical consent)at the Department of Urology, Icahn School of Medicine at Mount Sinai, New York, 10029. All necessary patient/participant consent has been obtained and the appropriate institutional forms have been archived.

**Table:** 

Patient	BX Gleason	Path Gleason	Pathology stage	Initial PSA
p1	4 + 4	4 + 4	T2	12.56
p2	4 + 5	4 + 5	NA (T3)	2.7
p8	4 + 4	4 + 3	T2	46

### Method details

#### EV extraction from serum and cell culture medium using nanoDLD and ultracentrifugation

##### Cell culture EV isolation

Human prostate cancer cell lines, 22RV1, purchased from American Type Culture Collection (ATCC) and maintained in RPMI 1640 cell culture medium (GIBCO). 22RV1 cell lines are supplemented with 1% antibiotic and were monitored till 80-90% cell confluency was achieved. The supernatant was then extracted and centrifuged at 300 x g at 4°C; the resulting cell pellet comprised of dead cells and cellular debris were then removed. The remaining supernatant is transferred into a new 50mL tube and further centrifugated at 2,000 × g, 4°C for 30 minutes allowing for larger vesicles and remaining cell debris to be pelleted and removed. The supernatant is then transferred into another 50mL tube and diluted till the total volume is 3/4 of volume of the tube. Sequentially, the solution is centrifugated at 20,000 × g, 4°C for 45 minutes followed by ultracentrifugation at 120,000 × g, 4°C for 2 hours (using Beckman coulter, thick wall polypropylene tube, Cat # 355642). The pellet derived from the ultracentrifugation is washed and resuspended in PBS followed by another ultracentrifugation at 120,000 × g, 4°C for 2 hours. Finally, the pellet is collected and resuspended in 100 ul of PBS and stored at −80C.

##### Serum derived EV isolation via ultracentrifugation

Blood from prostate cancer patients was collected via BD Vacutainer blood collection tubes and serum isolation was performed using serum separation tubes from Fisher Scientific (Cat.# 368016). 2-5 mL of the isolated serum was aliquoted and centrifuged at 2,000 × g, 4°C for 30 minutes. The supernatant is then transferred to a new 50 mL tube. To ensure the fluid volume is 3/4 of the total volume, 0.2um filtered PBS was added to the sample supernatant. The resulting solutions were centrifuged at 20,000 × g, 4°C for 45 minutes. Sequentially, the supernatant is ultracentrifuged at 120,000 × g, 4°C for 2 hours (using Beckman coulter, thick wall polypropylene tube, Cat # 355642). The pellet derived from the ultracentrifugation is washed and resuspended in PBS followed by another ultracentrifugation at 120,000 × g, 4°C for 2 hours. Finally, the pellet is collected and resuspended in 100 ul of PBS and stored at −80C.

##### Serum EV isolation via nanoDLD

Aside from the conventional methodology of EV isolation via ultracentrifugation, we have implemented an innovative method for serum EV isolation by utilizing the nanoDLD apparatus. To minimize non-specific adsorption, we have primed the chips using a 0.02 μm-filtered solution of 3% (w/v) bovine serum albumin (Sigma Aldrich) in phosphate buffer saline. Prefiltered (0.4 μm) samples were placed through the apparatus setting at G = 225 nanometer for 1 hour at Papp of approximately 5 bar. EVs in the range of 70-100 nanometers were primarily delegated into the bump fraction. Of note, smaller (<50 nm) particles remain in zigzag fraction of nanoDLD and are not collected for analyses. Rigorous characterization of UC and nanoDLD isolated EVs from various biofluids has been presented in our previous publications (Murillo et al., 2019; Smith et al., 2018).

##### Nanoparticle tracking analysis (size and zeta potential)

Prior to performing nanoparticle tracking analysis, samples collected were further diluted using Millipore DI water to a targeted concentration of 10^6^-10^7^ particles/mL. ZetaView was then utilized to evaluate the particle size concentration and zeta potential via the built-in EMV Zeta protocol. Nanoparticle tracking analysis results verify the presence of EVs in the bump fraction of the serum samples from the nanoDLD apparatus. Likewise, the detected EV concentrations appeared to be ∼2.6 to 3 times higher than that found in the input fractions.

##### Immuno-fluorescence co-localization analyses (Nanoview)

Briefly, canonical tetraspanin exosome markers CD81, CD9, and CD63 against the EV surface are arrayed on silicon chips. EV suspensions are incubated with the chips overnight. After incubation, chips are washed with PBS on a shaker and air dried. Captured EVs are detected using Single Particle Interferometric Reflectance Imaging Sensor technology.

#### TEM analyses

##### EV TEM analyses

Frozen EV pellet was brought to room temperature. Equal volumes of EVs and 3% Glutaraldehyde were mixed and kept at room temperature for 1 hr. Osmium tetraoxide was added to the EV solution and was kept at room temperature for 1 hr. The final EVs solution was transferred to formvar coated TEM grid and dried slowly. The grids are observed under the electron microscope at 80 kV. TEM grids are stored in the appropriate grid storage boxes for future use. Hitachi 7000 transmission electron microscope operating at 80 kV was used for imaging.

##### Immuno-gold labeling of EVs

Frozen EVs pellet was brought to room temperature. Equal volumes of EVs and 3% Glutaraldehyde were mixed and kept at room temperature for 1 hr. 2ul of EV pellet was transferred to formvar coated TEM grid (at least 2 grids were prepared for each sample). TEM grids were covered and dried at room temperature for 30 minutes. 100ul drops of PBS were transferred to Parafilm. Dried TEM grids were carefully washed by transferring them on top of the PBS drops with the help of forceps (this step is repeated 5 times). The grid is transferred to a 100ul drop of BSA (this step is repeated 5 times). Then, the grid is transferred on top of a 5ul drop of primary antibody (CD81) in a blocking buffer for 30 minutes. Transfer the grid to a washing buffer/blocking buffer for 5 minutes (this step is repeated 5 times). A drop of 5ul gold conjugated secondary antibody is transferred to the Parafilm. The grid is transferred (and covered) on top of the gold antibody drop for 30 minutes. Once completely incubated, the grid is washed by keeping on top of 100ul PBS solution for 3 minutes (this step is repeated 10 times). Finally, contrast the EVs on the TEM grid with osmium tetraoxide for 10 minutes. The grid is ready for TEM imaging.

##### RNA extraction, library preparation, and next-generation sequencing

Total RNA was extracted from the serum bump fraction of nanoDLD, serum EVs pellet from UC using the Total EV RNA and Protein Isolation Kit (Invitrogen 4478545). 50 μL EVs from bump fraction, 100-200ul serum EVs were resuspended in equal volume of ice-cold EV resuspension buffer. 2X denaturing solution was added to the final EVs solution on ice. Equal volume of acid- phenol:chloroform solution was added to each sample. The final solution was vortexed for 60 seconds and centrifuged at 10,000 x g. The top aqueous phase was carefully isolated without disturbing the lower organic phase. The top aqueous phase was transferred to the provided filter cartridge in collection tubes. Bound RNA was washed 3 times using the included wash solution. Finally, a preheated elution solution was used to elute the RNA in 100 μL volume. RNA was stored at −20°C until it’s RNA quality was assessed by bioanalyzer (Agilent 2100 Bioanalyzer, RNA 6000 Pico Kit, Agilent Technologies).

cDNA Libraries were prepared for small RNAs using the SMARTer smRNA-seq Kit for Illumina (Takara Bio 635030). A total of 18 cycles of PCR were carried out to obtain a good yield of cDNA from tissue, cells, and EVs. Final library quality was verified with Qbit and bioanalyzer. Negative (no RNA) and positive controls provided expected results. Next-generation RNA sequencing was performed using a HiSeq 4000 (Illumina), 100 base pair, single-end reads at the New York Genome Center.

##### Mass Spectrometry

Mass Spectrometry was conducted at Herbert Irving Comprehensive Cancer Center (HICCC) by Dr. Emily Chen, PhD. Each frozen cell pellet was homogenized by adding a pre-determined volume of lysis buffer (2% SDS/1X protease inhibitor/0.1M Ambic). Enhanced BCA Protein Quantification assay was used to determine the total protein amount from each sample. Proteins from 30μg of lysates were separated from SDS using micro S-trap columns (http://www.protifi.com/s-trap/) and digested on column by trypsin. Resulting peptides were labeled with TMT6plex isobaric reagent per sample and then combined for high pH reverse phase peptide fractionation. Thermo Orbitrap Fusion Tribrid Mass Spectrometer was used for MS/MS analysis (MS3 data acquisition method). Three technical replications were run per sample. Proteins from 20ul of exosome lysates were separated from SDS using micro S-trap columns (http://www.protifi.com/s-trap/) and digested on column by trypsin. Resulting peptides were speedvac dried for LC-MS/MS analysis. Thermo Orbitrap Fusion Tribrid Mass Spectrometer was used for MS/MS analysis. Global normalization based on total number of ms/ms spectra (PSM) acquired was applied to the MS data. Spectral counts were used for semi-quantitative analysis to compare protein abundance among different samples.

Proteome Discoverer software (version 2.1) was used to search the acquired MS/MS data against a human protein database downloaded from the UniProt website and generate TMT ratios. Positive identification was set at 5% protein FDR and 1% peptide FDR. Also, at least 1 unique peptide has to be identified per protein. A total of quantifiable proteins from this study is 5630 proteins. TMT ratios (each tag/common reference) were calculated by PD 2.1 and normalized by total peptide amount. Qlucore Omics Explorer package was used to perform statistical analysis. Ingenuity Pathway Analysis (IPA) was used to perform data mining.

Proteome Discoverer software (version 1.4) was used to search the acquired MS/MS data against a human protein database downloaded from the UniProt website. Positive identification was set at 5% protein FDR and 1% peptide FDR. Also, at least 2 unique spectra has to be identified per protein. Scaffold Proteome Software was used for post-database search processing. 422 proteins passed the filtering criteria and their expression profiles among these four samples were analyzed to identify differentially expressed proteins. Qlucore Omics Explorer Statistical Software was used to perform appropriate statistical analysis.

### Quantification and statistical analysis

#### Genome mapping

For quantification of gene expression, raw reads were aligned to the latest Ensembl GRCh38.p13 (GCA_000001405.28) using bowtie aligner (version 2.5.4b). FeatureCounts was then used to map the aligned reads to the GENCODE v26 primary gene annotation, including transcripts corresponding to ncRNAs such as lncRNA, miRNA as well as protein-coding RNA. To maximize recovery and minimize the noise, multimapping reads were quantified up to m = 10 and distributed using unique reads mapping distribution, as described in most recent best practices protocols.

#### Formal analysis

Data cleaning, filtering, and analysis were performed in R and under expressed genes or proteins with low or no counts across all samples of the similar phenotype were removed (at least one of the samples have CPM >10). Normalization via trimmed mean of M-values in edgeR ensures library sizes of all samples are scaled properly to minimize the influences of external factors. The limma package, originally designed for microarray data, performs linear modeling on normally distributed data. Thus, to accommodate for the non independent mean-variance relationship of RNA-seq data, the voom function assigns a precision weight derived from the library size and normalization factor of each sample itself to convert the raw counts to log2-CPM values. The log2-transformed counts minimize the changes in variance as the count size increases. Prior to examining differential expressions, we performed unsupervised clustering of samples to evaluate the similarities and dissimilarities between samples as well as across phenotypes of interest using the prcomp package in R. The result is reflected in the PCA plots.

Differentially expressed genes are discerned between 1) 22RV1 cell lines versus 22RV1 cell-line-derived EVs, and 2) Prostate cancer patient serum-derived EVs isolated using nanoDLD versus the EVs isolated using UC via the standard differential expression pipeline as illustrated in limma/edgeR packages. Results of the differentially expressed genes are represented in high-resolution heatmap as well as volcano plots made using pheatmap and ggplot2 packages. Likewise, differentially expressed proteins are discerned between 1) 22RV1 cell-line, 2) the EVs derived from the corresponding cell lines, and 3) patient serum EVs derived from UC versus those derived from nanoDLD.

Correlation analyses: Spearman Rho correlations were determined across cellular and EV genetic profiles as well as the proteomic profiles. Gene expressions were plotted in the x/y axis, where x/y axis are log2 (CPM), all RNA types were analyzed.

Biotype analysis: The gene biotype was recovered from the GTF annotation file for Ensembl GRCh38 (same as for alignment). Mapping resolution was kept as CDS with intron and exon annotation levels and combined to gene level when necessary. After differential expression quantification of gene biotype proportions, numbers and expression levels was taking into account. Thus, expressing gene biotype as (1) number of molecules per biotype (after lib. size adjustment) and (2) levels of expression using RPKM to adjust for gene/transcript length sizes.

Pathway Analysis: To effectively compare, not only enriched genes, but also against enriched proteins, pathway enrichment analyses are performed using the enrichR package in R. Specifically, we referenced databases including Kyoto Encyclopedia of Genes and Genomes (Versions: 2013, 2015, 2016, 2019, 2021), Gene Ontology Molecular Function (Versions: 2013, 2015, 2017, 2017b, 2018, 2021), Gene Ontology Cellular Component (Versions: 2013, 2015, 2017, 2017b, 2018, 2021), Gene Ontology Biological Process (Versions: 2013, 2015, 2017, 2017b, 2018, 2021), Reactome (Version: 2016), and WikiPathways (Version: 2019). Top 1500 enriched genetics and all of the proteomic signatures were used for the pathway analysis. Top 10 genomic and proteomic pathways from each database with FDR below 0.05 and at least three enriched genes present were selected. The overlaps across different cellular and EV datasets are outlined in the barplot made using the ggplot2 package in R. Similarly, we also performed the gene set enrichment analysis using our RNAseq result for hallmark cancer pathways. GSVA scores are generated per sample using the GSVA program in R. Wilcoxon test is then performed to identify significant GSVA scores across both cell and EV samples with a cutoff of 0.05 FDR. Of which, pathways with GSVA score differences greater than 0.1 across the cell and EV samples are then represented in a heatmap. Finally, upon the acceptance of manuscript our data and results will be uploaded to GEO and will be openly available.

## Data Availability

Data reported in this paper has been shared by the lead contact. This paper does not report original code. The code used is available on https://github.com/chentytina/22RV1_EV or upon request. Any additional information required to reanalyze the data reported in this paper is available from the lead contact upon request.

## Supplemental information

Document S1. Figures S1 and S2 and Tables S1–S4

Table S5. Raw RNAseq data for cells and EVs, related to Figure 4

Table S6. Normalized Transcriptomics data for cells and EVs, related to Figure 4

Table S7. Raw Mass Spec data for cells, related to Figure 3

Table S8. Normalized Mass Spec data for cells, related to Figure 3

Table S9. Raw Mass Spec data for cells, related to Figure 3

Table S10. Normalized Mass Spec data for cells, related to Figure 3
