# Supplementary material for: Extracellular vesicles carry distinct proteo-transcriptomic signatures that are different from their cancer cell of origin
Source: iScience. 2022 May 18;25(6):104414. doi: 10.1016/j.isci.2022.104414 (PMC9157216; doi:10.1016/j.isci.2022.104414)
Supplement: Document S1. Figures S1 and S2 and Tables S1–S4 [file mmc1.pdf]

**iScience, Volume *25***

## Supplemental information

# Extracellular vesicles carry distinct proteo-transcriptomic signatures that are different from their cancer cell of origin

**Tzu-Yi Chen, Edgar Gonzalez-Kozlova, Taliah Soleymani, Sabrina La Salvia, Natasha Kyprianou, Susmita Sahoo, Ashutosh K. Tewari, Carlos Cordon-Cardo, Gustavo Stolovitzky, and Navneet Dogra**

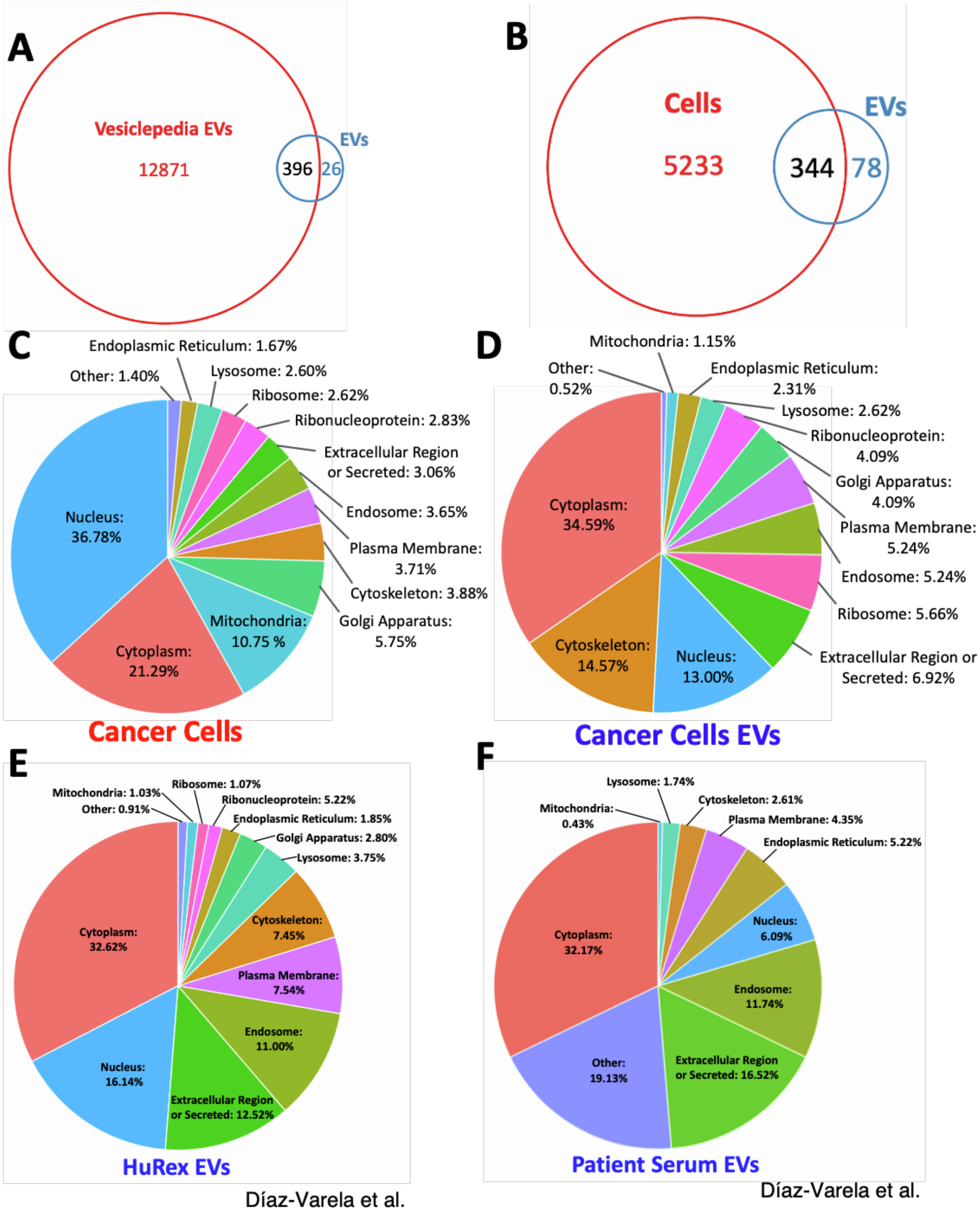


**Supplementary figure 1. Proteomic analysis of donor cells and their EVs, related to Figure 3.** (A) Comparative analyses of Vesiclepedia proteomic database of EVs with in-house proteomics of EVs isolated from prostate cancer cell lines, 22RV1. (B) Comparative analyses of EV and their donor cell proteins. (C) Subcellular location of donor cancer cell proteome. (D)

Subcellular location of cancer cell EVs proteome. Analyses of external proteomic data (from Diaz-Varela et al.) from human cord blood reticulocyte-derived (HuRex) exosomes (E) and human serum EVs (F).

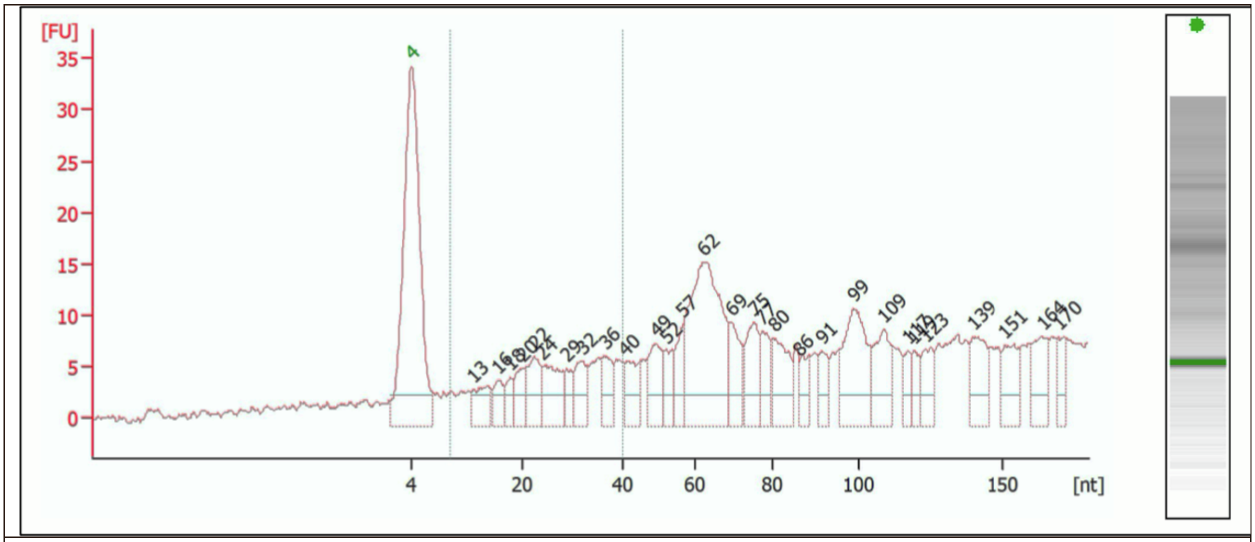


**Supplementary figure 2.. Small RNA analyses of EV RNA, related to Figure 4A.** Capillary electrophoresis shows diverse RNA types in EVs ranging from 13-200 nt. Peak at 4nt is a spike-in marker.

| Top EV RNA | | | | Interacting RNA and proteins | |
|---|---|---|---|---|---|
| Serum EVs (UC) | Serum EVs (nanoDLD) | 22rv1 EVs (UC) | | EV_RNA | interacting EV_protein |
| MT-RNR2 | TCTE1 | VTRNA1-2 | | VTRNA1-2 | IGF2BP2, SRSF1 |
| RNY4 | PEX5L | VTRNA1-1 | | VTRNA1-1 | IGF2BP2, SRSF1 |
| VTRNA1-1 | CCDC96 | RNY1 | | RNY1 | YBX1 |
| AC124068.2 | FLNA | MYOCOS | | SPEG | let-7 |
| RN7SL2 | CELF2 | RN7SL2 | | AC124068.2 | |
| RNY1 | SLPI | SPEG | | DPP9 | snhg16 |
| TCTE1 | DBN1 | AC124068.2 | | VTRNA1-3 | igf2bp2 |
| PEX5L | MT-RNR2 | DPP9 | | RNY4 | ybx1 |
| SLPI | AC124068.2 | VTRNA1-3 | | RNY3 | ybx1 |
| CCDC96 | AC127164.1 | RNY4 | | | |
| CELF2 | AC109466.1 | RNY3 | | | |
| FLNA | ADGRG6 | MIR4454 | | | |
| MYOCOS | KCNIP2 | MAP1LC3B | | | |
| DBN1 | NRXN3 | AL049796.1 | | | |
| MIRLET-7 | | | | | |

**Supplementary Table 1. Interacting RNA and proteins discovered in EVs, related to Figure 6D**: Top enriched RNA in EVs and complementary EV proteins.

| Unique EV signatures compared with Vesiclepedia | | | | | | |
|---|---|---|---|---|---|---|
| UMAD1 | H1-0 | MT4 | AL136295.1 | H1-4 | SYT4 | H2BC11 |
| H3-3A | EPRS1 | H4-16 | TRAFD1 | H1-5 | L1RE1 | GFP |
| DEUP1 | H2AZ1 | H1-10 | MACROH2A1 | YBX3 | CHTOP | |
| CD59 | H2AC11 | MVB12A | ATP5F1B | ATP5F1A | DYRK1B | |

**Supplementary Table 2. A list of ~6% (26) proteins, which are unique to EVs as compared with Vesiclepedia database, related to Figure 3.**

| Unique EV signatures as compared to donor 22RV1 cells | | | | | | | | |
|---|---|---|---|---|---|---|---|---|
| COL1A2 | GALNT3 | NLRP1 | SERPINA3 | RRAS | ITGA1 | FLG2 | MVB12A | VPS37B |
| FBN2 | DEUP1 | RELN | HRAS | NAGA | ACTA1 | MAST2 | ITCH | CALML5 |
| CORIN | CD59 | SNRPN | KRAS | FLG | HSPG2 | KRT26 | ZC3H10 | MKRN1 |
| AGRN | UBC | PPL | IGHA1 | AZGP1 | PKD1 | VPS37D | FAM193B | DICER1 |
| LYZ | TUBA1B | WWP2 | FN1 | DCT | LRP2 | NOSTRIN | SH3GL2 | DYRK1B |
| TNXB | HBB | QSOX1 | HPX | USP8 | PLAUR | PROM2 | LAT2 | TNFRSF11A |
| UMAD1 | SDCBP | IGSF3 | S100A8 | GFP | DSC1 | SNX33 | EPN3 | |
| VPS37C | HCK | FAM13A | ALPL | LGALS7 | TRIM26 | IGSF8 | SMOC1 | |
| EIF1AY | ABCA3 | CLDN7 | H2AC11 | MT4 | CEP55 | SH3KBP1 | THAP9 | |

**Supplementary Table 3: A list of 78 (22%) proteins, which are unique to EVs as compared with their donor cells, related to Figure 3.**

| | High in cells | | | | | | | High in EVs | | | |
|---|---|---|---|---|---|---|---|---|---|---|---|
| | Cells | EVs | cell_line | sCellsmbol | ensembl | label | | Cells | EVs | cell_line | sCellsmbol |
| 1 | 0.90455893 | 0.19969524 | 22RV1 | MT-TV | ENSG000002 | Cells | 1 | 0.10787277 | 0.83577806 | NF | MIR3591 |
| 2 | 0.88258295 | 0.23594825 | 22RV1 | RELN | ENSG000001 | Cells | 2 | 0.23874186 | 0.76553983 | CAF | MIR4508 |
| 3 | 0.88232726 | 0.13403299 | 22RV1 | MT-TK | ENSG000002 | Cells | 3 | 0.10341913 | 0.72830188 | 22RV1 | SCN4B |
| 4 | 0.86716709 | 0.23594825 | 22RV1 | MTRNR2L8 | ENSG000002 | Cells | 4 | 0 | 0.72424547 | CAF | MIR3591 |
| 5 | 0.85782672 | 0.23594825 | 22RV1 | MT-RNR1 | ENSG000002 | Cells | 5 | 0.2231927 | 0.71202652 | CAF | |
| 6 | 0.85390558 | 0.19969524 | 22RV1 | MT-TP | ENSG000002 | Cells | 6 | 0.10225728 | 0.68007096 | CAF | |
| 7 | 0.82778252 | 0.22552475 | 22RV1 | MT-TE | ENSG000002 | Cells | 7 | 0.18318049 | 0.66465689 | 22RV1 | ALDH1A3 |
| 8 | 0.82543984 | 0.09149175 | 22RV1 | MT-TL1 | ENSG000002 | Cells | 8 | 0.10341913 | 0.65589834 | 22RV1 | C1QA |
| 9 | 0.82097203 | 0.16205419 | 22RV1 | MTND6P3 | ENSG000002 | Cells | 9 | 0.21420798 | 0.64922753 | DKs.8 | REXO1L1P |
| 10 | 0.80695857 | 0.13403299 | 22RV1 | MT-TS2 | ENSG000002 | Cells | 10 | 0.2457349 | 0.63661343 | DKO.1 | REXO1L1P |
| 11 | 0.76945548 | 0 | 22RV1 | MT-TM | ENSG000002 | Cells | 11 | 0.10341913 | 0.61763141 | 22RV1 | ADAMTS7 |
| 12 | 0.7609625 | 0.09149175 | 22RV1 | IL21R | ENSG000001 | Cells | 12 | 0.22572863 | 0.61570638 | 22RV1 | COL4A2 |
| 13 | 0.74018145 | 0.16205419 | 22RV1 | MT-TD | ENSG000002 | Cells | 13 | 0.18112257 | 0.61124949 | CAF | RNU1-22P |
| 14 | 0.73819892 | 0.24578448 | NF | SNORD61 | ENSG000002 | Cells | 14 | 0.18112257 | 0.59728835 | CAF | |
| 15 | 0.73593289 | 0 | NF | SNHG15 | ENSG000002 | Cells | 15 | 0.15803075 | 0.5959489 | NF | MIR4488 |
| 16 | 0.73271508 | 0 | NF | | ENSG000002 | Cells | 16 | 0 | 0.58698526 | 22RV1 | AC006144.1 |
| 17 | 0.72623005 | 0 | 22RV1 | MT-TA | ENSG000002 | Cells | 17 | 0.22572863 | 0.58083029 | 22RV1 | YEATS2-AS1 |
| 18 | 0.72602815 | 0.18964622 | CAF | SNORD10 | ENSG000002 | Cells | 18 | 0.10341913 | 0.58004758 | 22RV1 | RF00019 |
| 19 | 0.71945043 | 0.22521572 | NF | RF00136 | ENSG000001 | Cells | 19 | 0.19106897 | 0.57269349 | NF | |
| 20 | 0.71736259 | 0.22521572 | NF | SNORD60 | ENSG000002 | Cells | 20 | 0.22005261 | 0.56903857 | DKO.1 | ESRG |
| 21 | 0.71509514 | 0.09149175 | 22RV1 | SNORA73B | ENSG000002 | Cells | 21 | 0.15150629 | 0.56823905 | 22RV1 | KHK |
| 22 | 0.70883989 | 0.09149175 | 22RV1 | MT-TR | ENSG000002 | Cells | 22 | 0.2231927 | 0.56510633 | CAF | MIR4488 |
| 23 | 0.70082795 | 0.16205419 | 22RV1 | MTRNR2L2 | ENSG000002 | Cells | 23 | 0.22572863 | 0.56509954 | 22RV1 | RF00019 |
| 24 | 0.69936543 | 0.12945368 | CAF | SNORD61 | ENSG000002 | Cells | 24 | 0.15150629 | 0.55637744 | 22RV1 | RNY1P11 |
| 25 | 0.69349697 | 0.22929403 | CAF | MIR199B | ENSG000002 | Cells | 25 | 0.10341913 | 0.55404632 | 22RV1 | POU4F2 |
| 26 | 0.68843128 | 0.11260786 | NF | SNHG1 | ENSG000002 | Cells | 26 | 0.24145446 | 0.54895051 | 22RV1 | RECQL4 |
| 27 | 0.68393622 | 0 | CAF | RNU5A-6P | ENSG000002 | Cells | 27 | 0.23874186 | 0.54839393 | CAF | |
| 28 | 0.68202929 | 0.11260786 | NF | SNORD59A | ENSG000002 | Cells | 28 | 0.2433541 | 0.54727303 | DKO.1 | HHLA1 |
| 29 | 0.68146062 | 0 | NF | SNORD18A | ENSG000002 | Cells | 29 | 0.22572863 | 0.54592607 | 22RV1 | IRX3 |
| 30 | 0.68038433 | 0.13403299 | 22RV1 | MT-TS1 | ENSG000002 | Cells | 30 | 0.15150629 | 0.54497293 | 22RV1 | GSTM4 |
| 31 | 0.67997326 | 0.19969524 | 22RV1 | | ENSG000002 | Cells | 31 | 0.2327935 | 0.54274096 | DKO.1 | SPINK9 |
| 32 | 0.67619049 | 0.09149175 | 22RV1 | MT-TN | ENSG000002 | Cells | 32 | 0.17357232 | 0.53773875 | DKs.8 | MUC4 |
| 33 | 0.672547 | 0 | 22RV1 | MT-TG | ENSG000002 | Cells | 33 | 0.15713755 | 0.53698179 | DKs.8 | MUC16 |
| 34 | 0.672547 | 0 | 22RV1 | MTATP8P1 | ENSG000002 | Cells | 34 | 0.19106897 | 0.5361891 | NF | MIR4508 |
| 35 | 0.66890434 | 0.16496753 | NF | SNORD58A | ENSG000002 | Cells | 35 | 0.15150629 | 0.53451746 | 22RV1 | FBXO45 |
| 36 | 0.66868806 | 0.19945597 | NF | SNORD21 | ENSG000002 | Cells | 36 | 0.24087803 | 0.53298657 | DKO.1 | TPTEP1 |
| 37 | 0.66656565 | 0.19969524 | 22RV1 | MT-TQ | ENSG000002 | Cells | 37 | 0.14980421 | 0.52891318 | CAF | |
| 38 | 0.66118964 | 0.1829835 | 22RV1 | MTCO2P12 | ENSG000002 | Cells | 38 | 0.24375953 | 0.52856958 | DLD.1 | TTN |
| 39 | 0.66034763 | 0 | 22RV1 | MT-TF | ENSG000002 | Cells | 39 | 0.24145446 | 0.52743161 | 22RV1 | PLEKHG2 |
| 40 | 0.65881276 | 0.09149175 | 22RV1 | MT-TH | ENSG000002 | Cells | 40 | 0.20841342 | 0.52560988 | DKO.1 | AL139397.1 |
| 41 | 0.65842688 | 0 | NF | SNORD12C | ENSG000002 | Cells | 41 | 0.24087803 | 0.52518248 | DKO.1 | TTN |
| 42 | 0.65829103 | 0.18964622 | CAF | RNU4-2 | ENSG000002 | Cells | 42 | 0.0996136 | 0.523759 | DKO.1 | MUC4 |
| 43 | 0.6552513 | 0 | NF | RNU5A-6P | ENSG000002 | Cells | 43 | 0.10341913 | 0.51837737 | 22RV1 | ADAMTS9 |
| 44 | 0.65441308 | 0.13403299 | 22RV1 | RF00096 | ENSG000002 | Cells | 44 | 0.12043901 | 0.51802033 | DKO.1 | MUC16 |
| 45 | 0.65375028 | 0 | NF | SNORD14D | ENSG000002 | Cells | 45 | 0.23874186 | 0.51781474 | CAF | RNU1-16P |
| 46 | 0.65375028 | 0 | NF | SNORD12B | ENSG000002 | Cells | 46 | 0 | 0.51771511 | 22RV1 | LCN10 |
| 47 | 0.65299107 | 0 | NF | RNU4-46P | ENSG000002 | Cells | 47 | 0.10225728 | 0.51486874 | CAF | MIR4532 |
| 48 | 0.65185636 | 0.19969524 | 22RV1 | MTCO1P12 | ENSG000002 | Cells | 48 | 0.23587318 | 0.51287483 | DKs.8 | MUC6 |
| 49 | 0.64922826 | 0.09149175 | 22RV1 | MT-TL2 | ENSG000002 | Cells | 49 | 0.22572863 | 0.51150508 | 22RV1 | FZR1 |
| 50 | 0.64917463 | 0.18964622 | CAF | SNORD21 | ENSG000002 | Cells | 50 | 0 | 0.51150508 | 22RV1 | CIDEC |

**Supplementary Table 4: A list of top 50 RNA enriched EVs and donor cells, related to Figure 4H.**

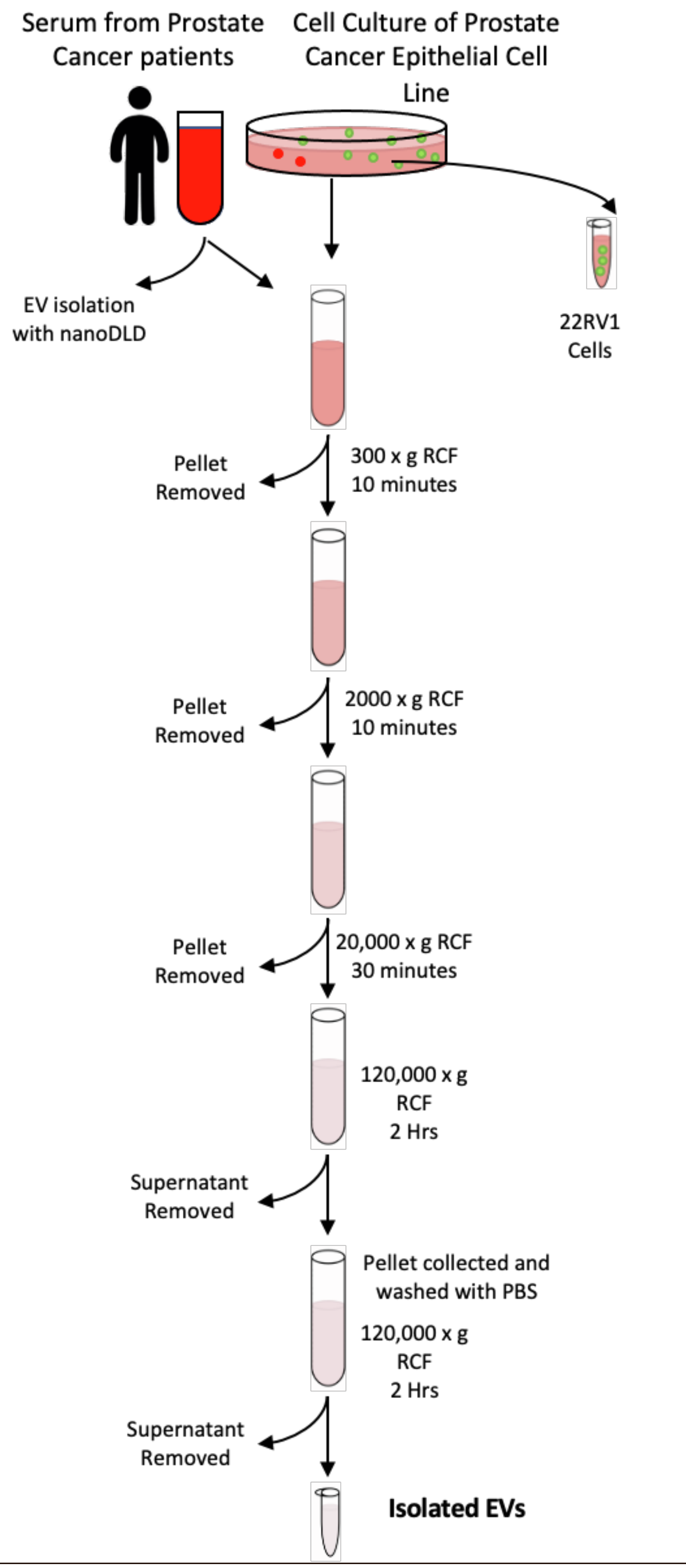


**EV isolation protocol, related to STAR Methods**
